# Short-term longitudinal participation trajectories related to domestic life and peer relations for adolescents with and without self-reported neurodevelopmental impairments

**DOI:** 10.1016/j.heliyon.2021.e06784

**Published:** 2021-04-13

**Authors:** Frida Lygnegård, Mats Granlund, Sabina Kapetanovic, Lilly Augustine, Karina Huus

**Affiliations:** a CHILD Research Group, Jönköping University, Jönköping, Sweden; b Swedish Institute of Disability Research, Jönköping University, Jönköping, Sweden; c School of Health and Welfare, Jönköping University, Jönköping, Sweden; d Department of Social and Behavioural Studies, University West, Trollhättan, Sweden

**Keywords:** Adolescent participation, Cluster analysis, Neurodevelopmental impairment, Participation trajectories, Person-based design, Self-report

## Abstract

**Background:**

With maturity and development, complexity in demands and roles change. As participation is often restricted in children with disabilities, this process might be delayed in adolescents. Investigating profiles of participation for adolescents with and without neurodevelopmental impairments could provide an understanding of which factors relate to high level of participation. The *aim* is to investigate trajectories of participation in everyday activities across clusters based on self-rated participation patterns in frequency of participation and perceived importance of activities related to domestic life and peer-related activities for adolescents with and without self-reported neurodevelopmental impairments.

**Methods and procedures:**

A prospective person-based cohort study design.

**Outcomes and results:**

Five typical trajectories were identified. Trajectories between clusters with high perceived involvement in peer relations were associated with sibling support and family communication. Self-reported neurodevelopmental impairments did not predict participation profiles at certain time points, nor movements between clusters when measuring self-reported attendance and importance in domestic life and in peer-related activities.

**Conclusion and implications:**

Perceived sibling support and family communication are important for predicting typical trajectories across clusters in frequency of attendance and the perceived importance of domestic life and peer relations. Type of impairment was less important in predicting typical trajectories.

## 1 Introduction

The first two decades of life are characterised by rapid growth and significant changes in physical, social and psychological development (World Health Organization, 2007). Particularly, adolescence has been emphasised as an important time period in human development, characterised by a sequence of emotional, physical and psychological maturity processes (Hilbrecht et al., 2008). During this stage of life, social relationships change. Although family is still the proximal part of adolescent development, peers become more important in terms of adolescent psychosocial functioning, including participation in different activities (Bornstein et al., 2013). This process of changes in expected life roles, from participating in home activities to participation in activities in the community, means exposure to more complex environments. Managing these developmental demands is linked to both growing competence and autonomy in adolescence and the support and affordances from the environment (World Health Organization, 2007). This process of changes in life role expectations and actual life roles might be delayed in adolescents with disability. Few studies have compared changes in participation in activities typical of adolescent life roles between adolescents with and without disability (Lygnegård, Augustine et al., 2018; van Gorp et al., 2019).

With increasing age, biological maturity and experience, life situations and thus also life roles change in both number and level of complexity. This includes participating in activities with a primary caregiver and social play activities outside the family, participation in peer relationships and social interactions related to school situations as well as leisure activities. In addition to developmental changes, emotional malfunctioning, i.e., distress and problematic behaviour (e.g., school truancy and misconduct) may increase during early and middle adolescence (Roeser and Eccles, 1998). How adolescents cope with emotional distress and participate in activities outside the home is dependent on their close relations with others, including parents, siblings, and peers (Noller, 2005). In that sense, the functioning of the individual should not be viewed in isolation but rather in the context of the family and peer systems. Therefore, understanding the role of the family environment and peers is crucial to understanding participation in activities outside the family setting as well.

Participation in everyday life can be viewed as an indicator of health (World Health Organization, 2007). Children and youth who actively participate in daily activities are provided with healthy developmental opportunities where skills important in adulthood can be acquired. Within the International Classification of Functioning, Disability and Health (ICF) and its version for children and youth (ICF-CY), participation is defined as ‘involvement in a life situation’ (World Health Organization, 2007); a definition also used in this study. Participation can be divided into two dimensions. First, the concept of being there, i.e., physically attending an activity. This can be measured objectively by measuring frequency of attendance, i.e., how often one attends a certain activity. The second dimension refers to the individual's own experience of involvement or degree of engagement and is not classified in the ICF-CY system. The experience of participation can be operationalised as the perceived importance the individual assigns to certain activities (Imms et al., 2017). Both dimensions are included in the present study, something which rarely occurs in research in this field (Shields et al., 2014).

Children with disabilities have the same interests and desires as all children (Cowart et al., 2004; Henry, 1998; Luttrop and Granlund, 2010). Nonetheless, the participation of children with disabilities is restricted, especially with regard to social activities outside the home environment. One reason is that they frequently have difficulties in peer relations such as handling interactions and resolving conflicts (Guralnick, 2010). In overcoming these difficulties, the family environment, i.e., the child's and adolescent's closest microsystem, becomes vital for learning interaction skills (Bronfenbrenner and Ceci, 1994). Participation restrictions vary between contexts and occur because of environmental factors and/or impaired body structures and functions (e.g., neurodevelopmental impairments) (World Health Organization, 2007).

One group of individuals known to have difficulties in social interactions are individuals with neurodevelopmental impairments (NDI), e.g., hyperactivity and specific language impairment. According to the Diagnostic and Statistical Manual of Mental Disorders, 5th edition (DSM-5) (American Psychiatric Association, 2013), neurodevelopmental impairments are defined as:

a group of conditions with onset in the developmental period. The disorders typically manifest early in development, often before the child enters grade school, and are characterized by developmental deficits that produce impairments of personal, social, academic, or occupational functioning. The range of developmental deficits varies from very specific limitations of learning or control of executive functions to global impairments of social skills or intelligence.

However, children and adolescents may have difficulties that can be described using criteria for NDI without having symptoms severe enough to prompt a diagnosis (Haller et al., 2014; Shankman et al., 2009). In this study, all adolescents' self-reported neurodevelopmental impairments were included. Earlier studies reveal conflicting results concerning participation restrictions and individuals with an NDI. While one study shows that adolescents with self-reported NDI are more likely to experience participation restrictions in school than their peers who do not self-report NDI (Carlberg and Granlund, 2018), other studies show no significant differences between adolescents with and without self-reported NDI regarding participation in domestic life and peer relations (Lygnegård, Augustine, et al., 2018). One explanation for these conflicting results is that the presence of an impairment is only one of several factors that may affect participation patterns or that the impact of impairment may vary depending on context. In this study, we investigate the participation patterns in peer relations and domestic life at two time points. Of special interest are typical trajectories between two time points, since they provide information about the stability in a person's functioning, which is discussed in earlier research (Almqvist, 2006; Bertills, 2019). Environmental and individual characteristics of children are analysed as possible explanatory factors in typical trajectories.

To understand children and adolescents' experiences of participation in a holistic inter-related manner, the use of person-oriented approaches has been suggested. Studying participation profiles, rather than participation in one specific activity or aggregating participation in different activities to a mean value, gives a broader perspective on children's participation (Bergman et al., 2003). Using a variable-approach with the same database, Lygnegård et al., 2018a, Lygnegård et al., 2018b showed that the factors explaining different levels of participation differed between the groups of children with and without self-reported NDI when studied at two time points approximately 1.5–2 years apart. Fewer factors were associated with the participation outcomes at the first time point for students with NDIs. This difference was less evident at the second time point, where more factors were related to participation frequency in domestic life and perceived involvement in school (Lygnegård, Augustine, et al., 2018). The similarities between the groups were thus stronger at the second time point. This may indicate a slower change in life role-related participation for adolescents with self-rated NDI. To gain more knowledge of these differences, a holistic and multidimensional approach where homogenous patterns can be identified should be applied. In doing so, the factors that potentially affect identified changes in participation patterns can be found (Bergman et al., 2003). Moreover, given that a profile approach is emphasised by the ICF-CY, yet is difficult to determine using the ICF-CY classification system (Simeonsson et al., 2010), a person-based longitudinal design as applied in this study might supplement the ICF-CY when studying changes in functioning over time.

The aim of this study is to investigate typical trajectories across clusters based on self-rated participation patterns in frequency of participation and perceived importance of domestic life and peer-related activities for adolescents with and without self-reported neurodevelopmental impairments. The following research questions were addressed:

- What typical trajectories can be found among cluster groups for frequency of attendance and perceived involvement in domestic life and peer relations between two time points?
- To what extent do individual and environmental factors predict typical trajectories?
- Are adolescents with self-reported neurodevelopmental impairments overrepresented in any of the trajectories?

## 2 Methods

### 2.1 Study design and setting

This is a person-based longitudinal cohort study using self-reported data from the first and third of five data collections within the ongoing Swedish multidisciplinary research program LoRDIA (Longitudinal Research on Development in Adolescence). The program is designed to follow adolescents in four municipalities in Swedish compulsory schools as well as compulsory schools for adolescents with intellectual disabilities (ID). These municipalities each have between 9,000 and 36,000 inhabitants and are geographically relatively close to one another. The municipalities were chosen as they represent variations in rural and urban density. Two of the municipalities (A and B) have a relatively high degree of internal school migration; adolescents from the minor municipality (B) usually attend senior high school in the somewhat larger municipality (A). Both municipalities are industrial and relatively small. Municipality C is somewhat larger than A and B and close to Sweden's second largest city, while D is smaller and more rural than A, B and C. The data collection started in 2013, when adolescents were in the 6^th^ and 7^th^ grades. Within the present study, wave 1 and wave 3 are referred to as time one (T1) and time two (T2).

### 2.2 Participants

The analytical sample consisted of N 916 adolescents (55% girls; n = 504) who participated in both the T1 and T2 data collections. The adolescents' mean age was 13.0 (±0.59) years at T1 and 14.34 (±SD = 0.64) at T2. Regarding ethnicity and family situation, 18% came from another country and 82% were living with both parents. Adolescent self-reports revealed that 17% (n = 154) (49% girls; n = 75) had self-reported a neurodevelopmental impairment such as attention deficit hyperactivity disorder (ADHD), autism, intellectual disability or speech or language impairment/communication disorder (American Psychiatric Association, 2013).

### 2.3 Instruments

Data for the present study was collected using paper-based questionnaires where answers were self-rated. At T1 adolescents with ID were given the opportunity to use an adapted form of the questionnaire. The questionnaire was adapted and tested to make the questionnaire accessible for adolescents with intellectual impairments (with, for example, more concrete language and fewer response options). These adaptations were based on strategies recommended for children and youths with cognitive impairments (Nilsson et al., 2012). Of the participating adolescents at T1, 142 used the adapted version. At T2, the same questionnaire with the same measures was used for all adolescents (See Table 1 for an overview of dependent and independent variables.). Indexes with only two variables had low reliability due to the restriction in scale and number of items.

**Table 1:** Dependent and independent variables.

Dependent variables	Cronbach's alpha	Items	Response scale
Frequency of participation in domestic life	0.54	*How often do you:* -help out at home? -do grocery shopping? -prepare a meal? -wash your clothes?	Three-point Likert scale: 1 = never; 2 = sometimes; 3 = often
Perceived importance of domestic life	0.62	*How important is it to:* -help out at home? -do grocery shopping? -prepare a meal? -wash your clothes?	Three-point Likert scale: 1 = no; 2 = not really; 3 = yes
Frequency of participation in peer relations	0.35	*How often do you:* -make new friends? -get along with friends?	Three-point Likert scale: 1 = never; 2 = sometimes; 3 = often
Perceived importance of peer relations	0.31	*How important is it to:* -make new friends? -get along with friends?	Three-point Likert scale: 1 = no; 2 = not really; 3 = yes
Independent variables	Cronbach's alpha	N items included in index and example of items included in the index	Response scale
Experience of time and self N items: 4	0.70	Compared to peers at the same age, I feel: My friends treat me as if I were much: Compared to most people my age, I look:	Three-point Likert scale: 1 = younger 2 = about the same age 3 = older
Stress N items: 2	0.44	How often do you face time pressure?	Three-point Likert scale: 1 = never 2 = sometimes 3 = often
Support from siblings N items: 4	0.86	If I argue with my parents, my sibling supports me. If I get into trouble, I can expect help from my sibling.	Four-point Likert scale: 1 = I don't agree at all 2 = I partly agree 3 = I mainly agree 4 = I entirely agree
Family communication N items: 23	0.79	Items related to parental control^1^, parental solicitation^2^ and child disclosure^3^: How often do your parents ask you to tell them about things going on in your leisure time? Do your parents always demand to know where you are in the evenings, who you meet with and what you do together?	Three-point Likert scale: 1 = mostly 2 = sometimes 3 = seldom or never
Frequency of attendance in school N items: 1	Only one item	Have you been truant a whole day from school this semester?	Three-point Likert scale: 1 = no, not once; 2 = sometimes; 3 = many times
Perceived importance of school N items: 3	0.62	E.g., Do you try to do your best at school?	Three-point Likert scale: 1 = mostly; 2 = sometimes; 3 = almost never
Self-reported neurodevelopmental impairment^4^ N items: 1		E.g., ADHD, autism, intellectual disability, ASD	Three-point Likert scale: 1 = mostly; 2 = sometimes; 3 = almost never

1 The extent to which parents require their child to ask for permission before going out and the extent to which they insist on having information on their children's whereabouts.

2 The extent to which parents actively seek information about what their children do.

3 The extent to which children spontaneously disclose information on their whereabouts (Kerr and Stattin, 2000).

4 American Psychiatric Association (2013). The variables used were originally used in scales developed, used and validated by Arvidsson (2013), Arvidsson et al. (2012), Kerr and Stattin (2000; Stattin and Kerr, 2000). Mean value was used and calculated in all analyses.

The variables used were originally used in scales developed, used, and validated by Arvidsson (2013), Arvidsson et al. (2012) and Kerr and Stattin (2000; Stattin and Kerr, 2000). Mean value was calculated and used in all analyses. The dependent variables, defining the cluster variables (Table 1), were indices representing the frequency of attendance as well as the perceived importance of activities related to domestic life and peer relations. Nine independent variables were used in the study (Table 1). Self-reported NDIs were also used as an independent variable. The selection of variables was informed by a) correlations between the variables and b) previous use of these body, activity and environment indexes in a cross-sectional cluster analysis study, as variables which were assumed to affect participation in domestic life and peer relations (Lygnegård, Almqvist et al., 2018).

### 2.4 Procedure

Data was collected within schools and the questionnaire took 50–90 min to complete. Members of the research team were present in the classrooms to provide support if necessary. For some of the adolescents with ID or adolescents who spoke another language at home, additional time (approximately 30 min) was required. Participants filled in the questionnaires in classrooms and could ask questions regarding, for example, the meaning of words. A few exceptions for adolescents with ID were made. For example, in one case a staff member of the research team visited an individual who was given the opportunity to fill in the questionnaire at home. This was an option provided by the student's caregiver since it was the student's own wish to take part in the data collection. The adolescents were offered refreshments while completing the form. Adolescents with ID or adolescents with difficulties in reading and writing were provided the option to have the questions read aloud to them in a separate room.

### 2.5 Ethical considerations

As one of the aims of the LoRDIA research program is to investigate mental health and drug misuse, a so-called passive consent method was used, since it was crucial to include adolescents from families where the parents or caregivers might not agree to their children participating in the study. Asking for active consent from caregivers with low socioeconomic status was therefore assumed to put the participation of adolescents who would be able to provide valuable information in line with the aim and scope of the research program at risk. All parents or caregivers were informed via written information sent home about the study, both in Swedish and their native language. At each of the data collection occasions, a member of the research team informed the adolescents about the meaning of the principle of informed consent as well as the possibility of withdrawing from the study at any time without stating reasons for this. Ethical permission for the LoRDIA research program, consent procedure and data collection procedure were approved by the Regional Research Review Board in Gothenburg, Sweden (No. 362-13; 2013-09-25), and additional approvals were obtained for wave II (2014-05-20) (T446-14) and wave III (2015-07-31) (T553-15).

### 2.6 Data analysis

Data was analysed by performing a hierarchical cluster analysis (Ward's method) for each of the two time points. Cluster analysis is a technique used for classifying cases into relative groups called clusters (StatisticsSolutions.com).

Prior to the cluster analysis a residue analysis was performed in order to identify residual cases (outliers). For both T1 and T2 a nine-cluster solution was chosen. These cluster solutions were chosen after considering factors such as degree of explained variance and the level of homogeneity within the clusters (Bergman et al., 2003). To optimise the cluster solutions, a RELOCATE procedure was run in which individuals (cases) were moved between clusters to optimise the homogeneity coefficient.^1^ Individual stability in pathways between clusters, termed trajectories, was investigated by performing an EXACON procedure in order to obtain information on more common trajectories (types) and uncommon trajectories (antitypes).^2^ A typical trajectory is a movement between clusters that occurs with a higher probability than expected (Bergman et al., 2003). These analyses were performed using the software ROPstat (Vargha et al., 2015). Statistical Package for Social Sciences (SPSS) version 25 was used to conduct multinomial logistic regressions to investigate predictors of typical and atypical patterns of participation trajectories in frequency of attending and perceived involvement in domestic life and peer relations.

## 3 Results

A nine-cluster solution was chosen for both T1 and T2, considering frequency of participation and perceived importance in domestic life and peer relations. The Explained Error Sums of Squares (EESS) were 64.76 and 67.74 for the nine-cluster solution after RELOCATE for T1. For T2, the EESS were 62.84 and 66.22 for the cluster solution after RELOCATE.^3^ The five most common trajectories were then analysed further. The descriptive information of clusters at T1 and T2 for which trajectories were found are presented in Table 2.

**Table 2:** Descriptive information on clusters at time 1 and time 2 for which typical trajectories were identified.

	Time 1	Time 2	Time 1	Time 2	Time 1	Time 2	Time 1	Time 2	Time 1	Time 2
Cluster	1	1	2	5	3	9	5	2	6	7
*N*	166	139	85	125	154	82	100	96	132	133
Female/male (*n*) within cluster	124/42	92/47	42/43	79/46	85/69	50/32	57/43	54/42	75/57	64/69
Self-reported NDI (*n*) within cluster	22	22	17	16	24	16	23	13	16	22
Female/male NDI (*n*) within cluster	18/4	9/13	7/10	13/3	10/14	8/8	13/10	7/6	4/12	8/14

### 3.1 What trajectories can be found across cluster groups between the two time points?

**Figure 1:**
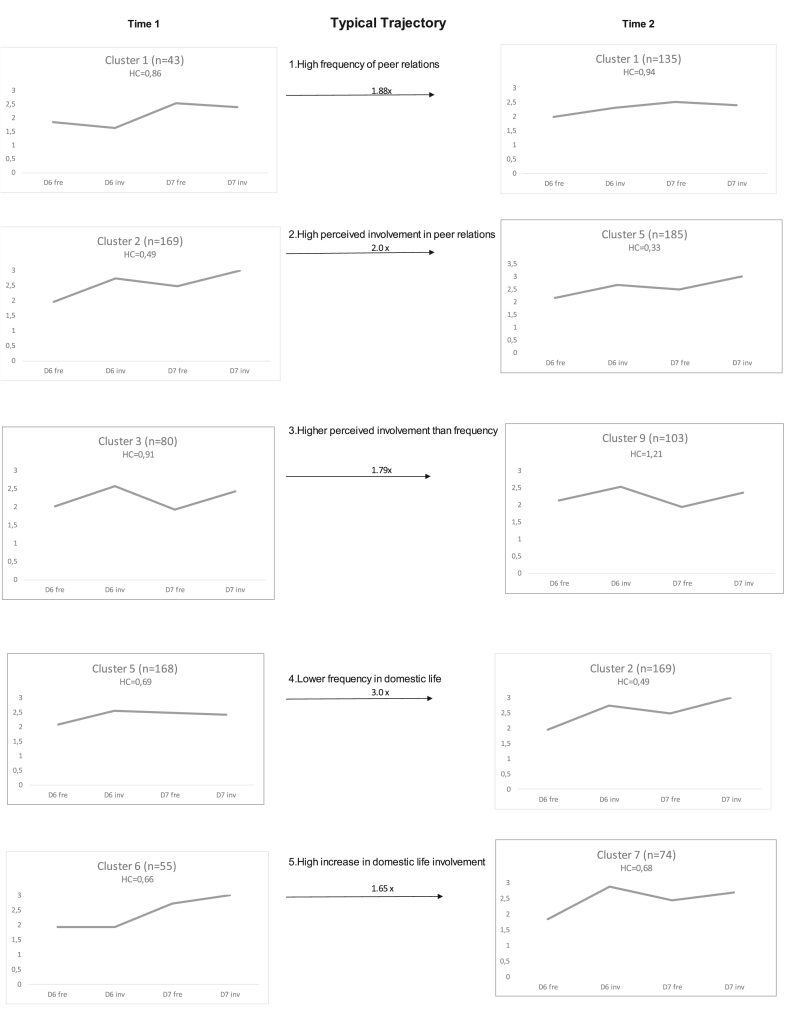
Overview of participation profiles (ICF-CY D-codes) at times 1 and 2 for clusters with typical trajectories. **D6 Fre** Frequency of involvement in domestic life, **D6 Inv** Perceived involvement in domestic life, **D7 Fre** Frequency of involvement in peer relations, **D7 Inv** Perceived involvement in peer relations. **HC** The Homogeneity Coefficient (HC) is the average of the pairwise distance within a cluster. Numbers attached to the end of the arrows indicate how many more times the flow was observed compared to what could be expected by chance. *P* ∗ ≤0.05, ∗∗ ≤0.01, ∗∗∗≤ 0.001.

Looking at attendance and involvement in domestic life and peer relations, cluster solutions were made at T1 and T2. The most typical trajectories were investigated further and five significant trajectories between T1 and T2 were identified. All five were characterised as typical trajectories, i.e., trajectories occurring more frequently than expected (with a total of 166 adolescents, i.e., 18% of the total sample). Thus, most of the individuals did not follow a more stable typical trajectory. The participation profiles at each time point, i.e., participants' ratings for frequency of participation and perceived importance in domestic life and peer relations at T1 and T2, informed the labelling of the clusters (illustrated in Figure 1).

As shown in Table 3, trajectory 1, labelled *High frequency of involvement in peer relations,* was the largest typical trajectory, with 49 adolescents (78% girls). Within this trajectory, 12% (n = 6) of the adolescents reported that they have an NDI. Adolescents in this trajectory were almost two times more likely to move from cluster 1 to the new cluster 1 at T2, a cluster characterised by higher involvement in peer relations. This trajectory is also characterised by a change in the participation profile between the two time points, with a high increase in the rating of perceived involvement in domestic life at T2.

**Table 3:** Overview of trajectories.

Trajectory	Label	Female/Male (n)	NDI Yes (n)	n within trajectory
1	High frequency of peer relations	38/11	6	49
2	High perceived involvement in peer relations	15/10	6	25
3	Higher perceived involvement than frequency	17/9	6	26
4	Lower frequency in domestic life	20/13	6	33
5	High increase in domestic life involvement	17/16	3	33

Trajectory 2, labelled *High perceived involvement in peer relations,* was the smallest typical trajectory, with 25 adolescents (60% girls), where 24% (n = 6) of the adolescents had a self-reported NDI. As shown in Figure 1, adolescents in this trajectory occurred two times more frequently than could be expected by chance in cluster 5 at T2. That means that it was twice as likely than expected that someone from cluster 2 at T1 moved to cluster 5 at T2. This trajectory is characterised by high ratings of peer relations at both T1 and T2 with a very similar participation profile at both time points. Adolescents in trajectory 3, labelled *Higher perceived involvement than frequency*, moved from cluster 3 at T1 to a similar cluster (cluster 9) at T2; both identified by higher perceived involvement than perceived frequency. In this trajectory, there were 26 adolescents, of whom 65% were girls and 23% (n = 6) had a self-reported NDI. For this trajectory there was a similar cluster profile at both T1 and T2, characterised by higher ratings of perceived importance of domestic life and peer relations than frequency of attending.

Trajectory 4, labelled *Lower frequency of involvement in domestic life*, consisted of 33 adolescents who were three times more likely to move from cluster 5 at T1 to cluster 2 at T2. There were 61% girls and 18% (n = 6) of the adolescents self-reported an NDI. This trajectory was characterised by a non-stable cluster profile from T1 to T2, with an increase for the ratings of both frequency and perceived involvement for domestic life activities and peer relations.

Adolescents in Trajectory 5, labelled *High increase in domestic life involvement,* moved from a cluster with lower frequency in domestic life to a cluster identified as higher in involvement but still low in frequency in domestic life. The total number of adolescents in this trajectory was 33 (52% girls). This trajectory had fewer adolescents with an NDI than the other trajectories (N = 3). A high increase in ratings for perceived involvement in domestic life and a slight decrease in the rating for the frequency of peer relations from T1 to T2 characterised this trajectory.

### 3.2 To what extent do individual and environmental factors predict the typical trajectories?

The associations between individual factors (i.e., the experience of time and self and perceived experience of handling stress) and environmental factors (i.e., sibling support and family communication) and participation trajectories are shown in Table 4.

**Table 4:** Multinomial logistic regression of associations between individual and environmental factors and the identified trajectories.^1^

	The experience of time and self		Stress		Sibling support		Family communication		Frequency of participation in school		Involvement in school	
	OR	CI 95%	OR	CI 95%	OR	CI 95%	OR	CI 95%	OR	CI 95%	OR	CI 95%
Trajectory 1 (Reference)	1	Ref	1	Ref	1	Ref	1	Ref	1	Ref	1	Ref
Trajectory 2	1.45	0.41–5.12	0.58	0.22–1.54	**0.32∗∗**	0.162–0.633	**26.62∗∗**	3.94–179.70	2.54	0.77–8.40	**11.23∗∗**	2.49–50.68
Trajectory 3	38.3	0.10–1.43	0.74	0.28–1.99	0.82	0.42–1.60	3.64	0.56–23.72	2.46	0.74–8.12	**5.61∗∗**	1.23–25.59
Trajectory 4	**6.02∗∗**	1.89–19.2	1.72	0.62–4.77	1.51	0.76–2.99	3.51	0.61–20.20	1.76	0.51–6.04	0.49	0.08–2.94
Trajectory 5	0.78	0.24–2.57	1.15	0.44–3.01	**0.49∗∗**	0.26–0.90	4.51	0.79–25.82	0.77	0.15–3.82	0.43	0.07–2.64
-2 Log Likelihood (p-value)	139	(<0.002)	48	(<0.001)	204	(<0.001)	301	(0.028)	46	(0.301)	93	(<0.001)
Nagelkerke Pseudo R^2^	0.096		0.456		0.113		0.064		0.032		0.120	

Note: ∗∗*p* < .001 ∗*p* < .05.

1 **Trajectory 1**: High frequency of peer relations; **Trajectory 2**: High perceived involvement in peer relations; **Trajectory 3**: Higher perceived involvement than frequency; **Trajectory 4**: Lower frequency in domestic life; **Trajectory 5:** High increase in domestic life involvement.

Adolescents with high ratings of the experience of time and self were six times more likely to belong to the trajectory *Lower frequency in domestic life* (#4) than to belong to the reference group *High frequency of peer relations* (#1). That is, the more mature one perceives oneself, the higher the probability of belonging to a trajectory with lower frequency and perceived involvement for domestic life activities, rather than belonging to a trajectory with high frequency of peer relations.

Furthermore, adolescents with high levels of sibling support were more likely to belong to the trajectory *High frequency of involvement in peer relations* (#1) than trajectory 2, thus *High perceived involvement in peer relations* and 5, and thus *High increase in domestic life involvement*. Adolescents who reported constructive communication in the family and perceived involvement in school were more likely to belong to the *High perceived involvement in peer relations trajectory* (#2). Perceived involvement in school was also positively associated with belonging to trajectory 3, thus *Higher perceived involvement than frequency*.

Adolescents with an NDI were evenly distributed within the typical trajectories. In one trajectory only three adolescents with an NDI were represented, versus six in the others. This could be a major difference, as it is half the amount present in the others. However, the fact that 17% of all adolescents with an NDI were represented in these five trajectories, and quite evenly distributed, indicates that this group pattern of participation is similar to those of typical development. For the typical group, 18% of all adolescents followed one of these five typical trajectories.

In sum, the typical trajectories labelled *High perceived involvement in peer relations* (#2) and *Higher perceived involvement than frequency* (#3) were relatively stable regarding the participation profile for self-reported participation in domestic life and peer-related activities. The trajectories *High frequency of peer relations* (#1) and *Lower frequency of involvement in domestic life* (#4) had a somewhat altered cluster profile at T2, where trajectory 1 had more similar ratings at T2 and trajectory 4 had a distinct increase in peer involvement at T2. Further, the trajectory *Lower frequency of involvement in domestic life* (#4) was associated with high ratings of time and self (i.e., feelings of being more mature compared to peers of the same age). Further, the trajectory *High perceived involvement in peer relations* (#2) was associated with high levels of sibling support, positive perceived family communication and perceived involvement in school. Belonging to the *High increase in domestic life involvement* (#5) trajectory was associated with experiencing higher levels of sibling support.

## 4 Discussion

Using a person-oriented design, this study investigated typical trajectories between cluster profiles of participation related to frequency of attendance and perceived importance of domestic life and peer-related activities. As only typical trajectories were identified, the clusters represented were a small share of the total population, namely 18% and 17% of the total NDI population. Results indicate that the participation profiles were relatively stable over a two-year period. Gender seems to have a more prominent role in predicting trajectories than NDI. There is an overrepresentation of girls in the typical trajectories except in trajectory #5.

The five most typical trajectory patterns between clusters at T1 and T2 were characterised by different degrees of involvement in peer relations and domestic life. Two of the trajectories, namely *High perceived involvement in peer relations* (#2) and *Higher perceived involvement than frequency* (#3), are characterised by having stable cluster profiles at T1 and T2 but vary in frequency of attendance and perceived involvement in peer relations in the cluster profiles. Adolescence consists of a time in life when individuals face challenges such as alterations in social relationships with both family and peers as well as physical and emotional changes associated with maturation (Inchley and Currie, 2014). The results could imply that changes relate to how often one attends activities and hence how often one is exposed to these. One might then argue that changes within expected life roles could lead to an increase or decrease of exposure to activities even though the pattern of activities remains the same.

The trajectories *Lower frequency of involvement in domestic life* (#4) and *High increase in domestic life involvement* (#5) are characterised by higher or lower involvement in domestic life in their cluster profiles from T1 to T2. A possible reason could be that individuals in trajectory 4 experience themselves to be more mature. The assumption drawn is then that an increase in domestic life involvement is due to increased maturation leading to more responsibilities taken at home. Trajectory 5 is characterised by lower odds in all factors. As this group was, both at T1 and T2, focused on high participation in peer relations, one can argue that this group is more peer-oriented than family- or school-oriented, even though they have an increase in domestic life involvement at T2.

Although the participation patterns seem to be relatively stable over time, as indicated by the stability in cluster formations (trajectories 2 and 3), some adolescents change their participation pattern between T1 and T2 (trajectories 1, 4 and 5). Individuals in trajectory 4 move to a cluster formation at T2 with higher involvement in domestic activities. One explanation for this might be that as one gets older and more mature family demands increase in relation to domestic life activities and domestic life activities are performed more on a routine base. Regarding peer relations, there is perhaps already a relatively established social network. Adolescents in the *High frequency of peer relations* trajectory (#1) move to a cluster profile characterised by higher involvement both in peer relations and domestic activity at T2.

Findings reveal that belonging to a certain typical trajectory was associated with varying individual and environmental factors. For example, higher perceived maturity (indicated by perceived ratings of time and self) was associated with belonging to the trajectory *Lower frequency of domestic life* (#4). This might imply that adolescents experiencing themselves to be more mature will over time be less involved in family life activities. Another factor that was related to trajectory was sibling support. Sibling relationships in adolescence have been proven to be important for psychosocial functioning (Noller, 2005). In the current study, when adolescents reported higher levels of support from their siblings, they were more likely to belong to the trajectory *High frequency of peer relations* (#1) than *High perceived involvement in peer relations* (#2) and *High perceived involvement in domestic life* (#5). This indicates that adolescents who perceive themselves as having a supportive relationship with their siblings also engage more in relations with peers. This parallels another study where results demonstrated that positive sibling relations seem to act as protective factors for social competence (Buist and Vermande, 2014). Arguably, support from peers could be associated with a decrease in the need of sibling support, i.e., spending time with siblings is okay but socialising with peers is prioritised. It is likely that individuals belonging to trajectory 2 and 5 belong to supportive families where explicit support from siblings is not as important, which might in turn lead to a social profile more directed towards peers. A safe connection to parents means that you can but do not always choose to talk to your parents, which is supported by results from this study where family communication was associated with belonging to the trajectory *High perceived involvement in peer relations* (#2). This trajectory had a stable cluster profile when time points were compared. Parental knowledge of their adolescents' activities as well as open communication between parents and their adolescent children is often considered a protective factor in adolescents' psychosocial development (Kapetanovic et al., 2019; Racz and McMahon, 2011). When parents have information about their adolescents' daily activities, they may give advice and guidance and serve as role models for adolescents' friendship relations (Engels et al., 2002). Thus, effective investment in peer relationships is particularly feasible and stable over time when adolescents have positive experiences of family relations. Trajectory 2 has a high probability to experiencing high school involvement, which might imply that individuals in this trajectory function well in everyday life activities.

Results also indicate that the more mature one feels, the higher the probability of belonging to the trajectory *Lower frequency in domestic life* (#4). This was somewhat surprising, given the assumption that the more mature one perceives oneself, the more frequently one takes part in domestic life activities, in contrast to what these results indicate. The result may rather indicate that adolescents who perceive themselves as more mature spend more time with peers and perceives domestic life activities as more important.

In sum, factors based on the perceived environment in the family are more prominent in predicting trajectories in participation in domestic life and peer relations than individual factors such as type of impairment. Adolescents with self-reported NDIs were not overrepresented in any of the trajectory groups, and they were very few.

Most adolescents did not follow a typical trajectory between clusters. This result indicates that participation profiles are relatively unstable for most adolescents. This supports earlier studies reporting that adolescence is a period of change. However, for a larger proportion than expected, namely 18%, clear pathways were found. These pathways were fairly stable over the two-year period. Moreover, they are characterised by fairly high levels of participation in domestic and peer involvement. Trajectory 1 does perhaps have more moderate participation, and trajectory 3 seems to be more home-oriented than peer-oriented, with lower participation in peer activities. Nonetheless, most of these clusters and trajectories are moderate to high in their level of participation.

### 4.1 Methodological considerations

#### 4.1.1 Limitations of the study

In this study the choice was made to study the most typical trajectories with the consequence that the less typical (atypical) trajectories were not studied, which should be considered a weakness. This is also why only 18% of the total population follow typical trajectories. This means that adolescents with an NDI do not appear in typical trajectories more frequently than expected, in comparison to adolescents without an NDI, with the implication that the trajectories adolescents with an NDI take remains a question that should be further investigated. The data used in the current study is based on self-ratings and it has not been possible to determine whether adolescents have a confirmed diagnosis or not. This should be taken into consideration when interpreting the results of this study. However, the use of self-reports makes it possible to capture adolescents' own opinions about their everyday situation, regardless of whether or not they have a diagnosis; individuals who report subthreshold difficulties (under the threshold for the diagnostic criteria) may experience difficulties affecting everyday functioning (Shankman et al., 2009).

In addition, in some cases the participation outcomes (the indices for frequency and perceived involvement in domestic life and peer relations) had relatively low internal consistency, which could arguably be a consequence of the complexity of the participation concept as well as the complexity of measuring participation. The homogeneity coefficient was above 1 in some of the clusters at T1 (cluster 9) and at T2 (clusters 6, 8 and 9), which is probably also a consequence of the relatively low internal consistency in some of the outcome variables in the cluster analysis. This study uses a total population-based sample which should be considered a strength. Adolescents with an NDI were, however, very few in relation to the total sample, which should also be taken into consideration when the interpreting the results of the current study, since this might lessen the generalisation potential of the results.

## 5 Conclusion

Self-reported participation is relatively stable when measuring two points in time approximately 2 years apart. Type of impairment did not predict participation trajectory. Environmental factors such as family communication are more important predictors for the identified typical trajectories, indicating that interventions aiming to enhance participation should be directed towards the persons surrounding the individual, including siblings.

## 6 Practical implications

When designing interventions aiming at increasing participation in domestic life and peer relations for adolescents with and without neurodevelopmental impairments it is important to include environmental factors such as sibling support and communication patterns in the family. This implicates not only the importance of an interdisciplinary approach but also the importance of identifying factors that affect participation and which are malleable by intervention. Family functioning is important for the longitudinal trajectories of participation in adolescents. It also implies that professionals need to involve family in data collection, not only regarding the adolescent but also aspects of family life. Having a self-reported neurodevelopmental impairment was not strongly related to type of participation trajectory, which indicates the need to assess the proximal environment independent of disability status of the adolescent.

## Declarations

### Author contribution statement

Frida Lygnegård: Conceived and designed the experiments; Performed the experiments; Analyzed and interpreted the data; Contributed reagents, materials, analysis tools or data; Wrote the paper.

Mats Granlund, Lilly Augustine, Karina Huus: Conceived and designed the experiments; Analyzed and interpreted the data; Wrote the paper.

Sabina Kapetanovic: Contributed reagents, materials, analysis tools or data; Wrote the paper.

### Funding statement

This work was supported by a combined grant from four Swedish research foundations: the 10.13039/501100004359Swedish Research Council (VR); the Swedish Research Council for Health, 10.13039/501100006636Working Life and Welfare (FORTE); Sweden’s Innovation Agency (VINNOVA); and the 10.13039/501100001862Swedish Research Council Formas (259-2012-25), as well as the Sävstaholm Foundation (ST-2014-023) and the Sunnerdahl Disability Foundation (22/17).

### Data availability statement

Data will be made available on request.

### Declaration of interests statement

The authors declare no conflict of interest.

### Additional information

No additional information is available for this paper.

## Footnotes

1 The homogeneity coefficient is the average of the pairwise distances within a cluster and measurers the degree of proximity (similarity or dissimilarity) between cases within the clusters. The lower the coefficient (preferably less than 1), the better the homogeneity within a cluster (Bergman et al., 2003).

2 A *type* can be defined as an expected movement between clusters and time points. An *antitype* can be defined as the most infrequent movement between clusters at the two time points.

3 The EESS should be 2/3 of the unexplained variance within the cluster solution (Bergman et al., 2003).
